# Report of Committee on State Medicine

**Published:** 1884-09

**Authors:** 


REPORT OF COMMITTEE ON STATE MEDICINE.—*Mr. President and Gentlemen:*—Your committee has the honor to report as follows: It has taken into consideration as coming within the scope of the resolution under which it was appointed, the following subjects:

First: The question of confidential communications. Second: The state of the laws regulating the supply of anatomical material. Third: The laws regulating medical education. Fourth: The laws regulating the relations of the insane to the community. Fifth: The question of suppressing indecent quack advertisements. Sixth: The question of practicing under an alias.

I. There is no statute regulating the relations of physicians and patients, as regards confidential communications, and the committee would recommend that the Society use all its efforts to secure the passage of the act now in force in New York, which is as follows: "No practitioner of medicine, surgery or midwifery shall be permitted to disclose any information which may be obtained from patients for purposes of diagnosis, prognosis, or treatment."

II. In regard to the supply of anatomical material, the committee would recommend the act proposed by the Chicago Demonstrators' Association as being adapted to meet the emergency.

III. In regard to the question of medical education, the committee believes that no degree of Doctor of Medicine shall be deemed valid, which has been conferred by any medical college on any one, who shall not have been previously examined as to his general education by a committee of the medical society of the school of practice, which said student proposes to follow; such committee to be chosen from members of the society not connected with any college; the certificate of the society to constitute evidence of such examination. The committee also believes that no medical college should hereafter be incorporated in this State, unless the same has an endowment fund of $150,000, free of all claim. The committee believes that no person should be hereafter permitted to enter upon the practice of medicine unless he shall have been previously examined by a board of examiners on Anatomy, Chemistry, Obstetrics and Gynæcology, Surgery, Practice of Medicine, Hygiene and State Medicine, Psychiatry, Therapeutics, Materia Medica and Pharmacy. This board of examiners should be chosen from the various schools of practice in proportion to the numbers of practitioners of each school. Five of these should be chosen by the various State medical societies, none of whom should in any way be connected with the medical colleges. The President and Secretary of the State Board of Health should be, *ex-officio*, members of this board. The surgeons of the *Army*, *Navy* and *Marine Hospital Service* should be exempted from the provisions of this act.

IV. The laws respecting the insane are in a very bad state and require revision in several particulars. By the present system of trying insanity—as if it were a crime—the insane are deprived of that early treatment which is their due, and family secrets become the property of the public. The judges often give local politicians the right to select private juries for the trial of friends who have become insane. A better law than the existing one would be one, which would not arouse public prejudice too much, and at the same time do away with the present unnecessary publicity. The committee believes that no person should be declared insane, and committed to a hospital for the insane, unless after the finding of a commission, appointed by a Judge of a Court of Record. Such commission should be composed of two physicians and one lawyer. This commission should have the power to take testimony and send for witnesses. The finding of the committee should be approved by the Judge of the Court of Record appointing it, and the person whose insanity is questioned should have the right to be represented by counsel chosen by the court, if he so desire. No physician should be appointed to such commission who has not been ten years in the practice of his profession and is not in good standing in the school of medicine to which he belongs; said good standing to be certified to by a medical society of the county in which he resides. At least one of the physicians should be an alienist, and his qualifications should be determined by the fact that he has been one or more years physician in a hospital for the insane, or has other special qualifications. Evidence of these qualifications must be submitted to the State Board of Health, which should issue a certificate that the applicant is a qualified alienist. No lawyer should be appointed to such commission unless he has been ten years in the reputable practice of his profession. No person should be discharged from any institution for the insane unless by a formal legal process, setting aside the finding which determined his insanity, or upon the application of friends, who should then be required to give such bonds, based on real estate, as the Judge of the Court of Record—to whom all such applications should be referred—shall deem necessary for the security of the public; but no person, not deemed recovered from insanity by the superintendent, should be discharged from any institution unless his or her friends have given bonds for their pecuniary responsibility for the results of his or her acts. The committee believes that no Justice of the Peace should permit any insane person or idiot to go at large, who has ever been brought before him on a criminal charge, but should straightway commit such lunatic or idiot to await the action of a commission, for which he should make immediate application to the Judge of the nearest Court of Record. In case such action is neglected, such Justice of the Peace should be held pecuniarly responsible for the results of such lunatic's or idiot's actions. All State institutions for the insane, and county institutions containing more than one hundred patients, should be placed under the control of the State Board of Charities, which should have the powers now delegated to trustees and other supervisory officials, connected with all such institutions. It should be the duty of each medical society of every county to appoint from its members gentlemen, qualified to act on commissions of lunacy. One representative, who with a similar representative from the bar association of the county, should constitute an inspection committee, which should examine monthly every institution for the insane and each recently admitted patient, and report the result of such examination to the State Board of Charities, which should take measures to remedy any abuses which might exist, and should, on the representation of such committee, transfer an insane person from a county to a State institution. All expenses of this inspection should be paid by the county.

No act which is the offspring of disease should be regarded as a crime. Any defense of insanity for crime should be allowed to be raised only at or before indictment, and the person, on whose behalf such a plea is raised, should be instantly transferred to a State institution for the insane and there kept under surveillance until his trial. The question of his insanity should be tried first before a jury composed of one-third physicians, qualified to act on lunacy commissions, one-third lawyers of equal competency, and one-third of citizens drawn as jurymen, as in ordinary criminal trials; but no one should be competent to serve on such a jury who has a prejudice against insanity as a defense for crime. If such jury find the prisoner insane, he shall be committed to a State institution for the insane for two years, and, at the expiration of that time, if the superintendent deem him recovered, the finding of the jury should be set aside by a lunacy commission, chosen in the way already mentioned. If the jury fail to find him insane, he shall stand his trial for the crime, and should not be allowed to plead insanity as a defense. No medical or legal officer of an institution for the insane should be qualified to sit upon any lunacy commission while he retains his office.

V. The indecent quack advertisements can be summarily dealt with in the manner prescribed in Pennsylvania. The Pennsylvania act is as follows: "Any person who shall publicly advertise in any way treatment of venereal disease, of youthful indiscretion, prevention of conception, of impotence, of self-abuse, or treatment of urinary disease, shall be deemed guilty of a misdemeanor, punishable by fine or imprisonment, or both."

VI. The committee also believe that any person convicted of practicing under an alias should be prohibited from future practice in this State.

Respectfully submitted,

JAS. G. KIERNAN, M. D., Chairman,
E. INGALLS, M. D.,
O. C. DEWOLF, M. D.,
Committee.

